# Comparison of 3T and 7T magnetic resonance imaging for direct visualization of finger flexor pulley rupture: an ex-vivo study

**DOI:** 10.1007/s00256-024-04671-x

**Published:** 2024-04-12

**Authors:** Thomas Bayer, Lilly Bächter, Christoph Lutter, Rolf Janka, Michael Uder, Völker Schöffel, Frank W. Roemer, Armin M. Nagel, Rafael Heiss

**Affiliations:** 1 Institue of Radiology, Universitätsklinikum & Friedrich-Alexander-Universität Erlangen-Nürnberg (FAU), Erlangen, Germany; 2 Klinikum Fürth, Institute of Neuroradiology and Radiology, Fürth, Germany; 3 Department of Orthopedics, University Medical Center, Rostock, Germany; 4 Department of Sports Orthopaedics, Sports Medicine, Sports Traumatology, Klinikum Bamberg, Bamberg, Germany; 5 Department of Orthopedic and Trauma Surgery, Friedrich Alexander Universität Erlangen-Nürnberg, FRG, Erlangen, Germany; 6 Section of Wilderness Medicine, Department of Emergency Medicine, University of Colorado School of Medicine, Aurora, CO USA; 7 School of Health, Leeds Becket University, Leeds, UK; 8 School of Medicine, Chobanian & Avedisian Boston University, Boston, MA USA; 9 Division of Medical Physics in Radiology, German Cancer Research Centre (DKFZ), Heidelberg, Germany

**Keywords:** Finger flexor pulleys, High field MRI, 3 Tesla, 7 Tesla, Climbing

## Abstract

**Objective:**

To compare image quality and diagnostic performance of 3T and 7T magnetic resonance imaging (MRI) for direct depiction of finger flexor pulleys A2, A3 and A4 before and after artificial pulley rupture in an ex-vivo model using anatomic preparation as reference.

**Materials and Methods:**

30 fingers from 10 human cadavers were examined at 3T and 7T before and after being subjected to iatrogenic pulley rupture. MRI protocols were comparable in duration, both lasting less than 22 min. Two experienced radiologists evaluated the MRIs. Image quality was graded according to a 4-point Likert scale. Anatomic preparation was used as gold standard.

**Results:**

In comparison, 7T versus 3T had a sensitivity and specificity for the detection of A2, A3 and A4 pulley lesions with 100% vs. 95%, respectively 98% vs. 100%. In the assessment of A3 pulley lesions sensitivity of 7T was superior to 3T MRI (100% vs. 83%), whereas specificity was lower (95% vs. 100%). Image quality assessed before and after iatrogenic rupture was comparable with 2.74 for 7T and 2.61 for 3T. Visualization of the A3 finger flexor pulley before rupture creation was significantly better for 7 T (*p* < 0.001). Interobserver variability showed substantial agreement at 3T (κ = 0.80) and almost perfect agreement at 7T (κ = 0.90).

**Conclusion:**

MRI at 3T allows a comparable diagnostic performance to 7T for direct visualization and characterization of finger flexor pulleys before and after rupture, with superiority of 7T MRI in the visualization of the normal A3 pulley.

## Introduction

The incidence of finger pulley rupture has increased in recent years [1]. These injuries are among the most common trauma in recreational and professional athletes in the new Olympic discipline sport climbing [2–4]. The annular pulleys (A1 proximal to A5 distal) are localized retinacular fibrous ligaments, reinforcing the flexor tendon sheaths on the palmar side of the fingers [5]. Almost exclusively affected by injury are the ligaments in the vicinity of the proximal interphalangeal joints (PIP) (i.e. A2, A3 and A4 pulley) [1]. This may happen, whenever high impact forces yield to overloading on an inflected finger pulley, which can also occur in non-climbers [6]. A correct diagnosis of pulley rupture is mandatory for therapy planning, as the pulleys are highly relevant for unrestrained finger flexion [6]. Non-treated pulley lesions may cause chronic inflammation and a reduction of the range of motion, eventually leading to contractures [7].

Direct visualization of finger pulley rupture is challenging using computed tomography, ultrasound (US) or MRI, because of the small size of the involved anatomic structures [8]. The particularly small A3 pulley could not be reliably visualized directly in several studies [8, 9]. Therefore, classic diagnostic concepts apply indirect US or MRI, which relies mainly on measurement of a rupture dependent, increased distance between finger flexor tendons and adjacent bone, also referred to as the bowstringing sign [8]. However, this indirect approach has shortcomings regarding the reproducibility and differentiation between isolated and combined pulley ruptures [10].

High-field MRI at 3 Tesla (T) and in particular ultrahigh-field MRI at 7T may allow optimized visualization of small anatomical structures with improved diagnostic performance in musculoskeletal imaging [11]. The signal-to-noise ratio (SNR) increases supralinear with the magnetic field strength (Bo) [12]. This SNR gain may be used to increase spatial resolution and to reduce acquisition time [13], which allows clinical application of direct imaging techniques for further MRI diagnostics of pulley ruptures. Studies of Goncalves-Matoso and Guntern examined direct pulley imaging at 3T for the functionally most relevant and anatomically largest A2 pulley [7, 14]. An ex-vivo study by Heiss et al. showed the possibility at 7T, to characterize all A2, A3 and A4 pulley lesions including rupture morphology, allowing direct diagnosis particularly also for the small A3 pulley and detection of potential associated rupture complications [15]. However, in contrast to 3T MRI, 7T installations are not widely available and examinations are hardly economically feasible. Therefore, we aimed to compare image quality and diagnostic performance of 3T and 7T MRI for direct depiction of finger flexor pulleys A2, A3 and A4 before and after artificial pulley rupture in an ex-vivo model using anatomic preparation as reference.

## Methods

### Preparation of the specimens

Thirty fingers from ten non-embalmed, paired hands were obtained from 3 female and 2 male cadavers donated to the Institute of Anatomy 1, Friedrich-Alexander-University Erlangen-Nuremberg, Germany. IRB (Institutional Review Board) approval was obtained from Friedrich-Alexander-University Erlangen-Nuremberg, Germany (260_15 Bc). All participants signed an institutionally approved informed-consent document at time of life for being a body donor. Donors had a mean age of 77.4 years (range, 55–94 years old) at the time of death. The forearms were harvested within 2 days after death and were stored at -5°C. The preparation of each specimen was performed after de-freezing according to the protocol described by Schöffl et al. [16]. The tendons of the flexor digitorum superficialis (FDS) and flexor digitorum profundus (FDP) muscle were prepared proximal of the fingers and were intersected at the myotendinous junction at the forearm. The index finger, middle finger and ring finger were extra-articulated. At that timepoint pre-rupture MRI was performed (see below). Subsequently, two Schanz's screws were placed within the proximal phalanx for later fixation in the loading apparatus for pulley rupture creation as described previously [16, 17].

### Pulley rupture creation

Fingers were placed in the crimp grip position [17] (which is a typical climbing position for maximum power transmission) in the loading apparatus. The finger flexor pulley ruptures were achieved after loading the FDS and FDP tendon of the fingers using an isokinetic loading device as described before [10, 18]. The flexor tendons were connected in series with the force transducers and the electric cage motor. Thus, we were able to increase the forces in the flexor tendons while the finger remained stationary, performing a concentric movement. Loading of the tendons continued until failure, either due to pulley rupture (ideally with an audible bang like often appearing in climbing) or any other failure mechanism (fracture of bone, tendon rupture, failure at the tendon-clamp interface) [16].

### Magnetic resonance imaging

MRI examinations were performed before and after iatrogenic rupture creation on a 7T system (Magnetom Terra, Siemens Healtheneers®, Erlangen, Germany) using a prototype 1-channel transmit/16-channel receive wrist radiofrequency coil (Rapid Biomedical GmbH, Rimpar, Germany) and on a 3T system (Magnetom Skyra, Siemens Healtheneers®, Erlangen) using a dedicated 8-channel wrist coil (Siemens Healtheneers®, Erlangen, Germany). MRI examinations with both field strengths were performed before and after artificial pulley injury. Fingers were placed in a cast with 30 degrees flexion within the PIP joint. A force of 10 N was applied by connecting a weight of 0.5 kg through a pulley rope system to the FDS and FDP tendon with equal distribution to both tendons [10]. At 7T a transverse T1-weighted sequence (total acquisition time, 4 min 46 s; echo time, 17 ms; repetition time, 700 ms; flip angle, 90°; resolution, 0.2 × 0.2 × 1.0 mm), a T2-weighted sequence (total acquisition time, 6 min 32 s; echo time, 68 ms; repetition time, 5000 ms; flip angle, 177°; resolution, 0.2 × 0.2 × 1.0 mm), and a PD-weighted sequence (total acquisition time, 6 min 32 s; echo time, 14 ms; repetition time, 5640 ms; flip angle, 177°; resolution, 0.2 × 0.2 × 1.0 mm) were acquired. At 3T a transverse T1-weighted sequence (total acquisition time, 8 min 49 s; echo time, 17 ms; repetition time, 700 ms; flip angle, 90°; resolution, 0.2 × 0.2 × 1.0 mm), a transverse T2-weighted sequence (total acquisition time, 6 min 20 s; echo time, 73 ms; repetition time, 5000 ms; flip angle, 170°; resolution, 0.2 × 0.2 × 1.0 mm), and a transverse PD-weighted sequence (total acquisition time, 6 min 29 s; echo time, 15 ms; repetition time, 5640 ms; flip angle, 177°; resolution, 0.2 × 0.2 × 1.0 mm) were acquired. The total acquisition time was 17 min 50 s at 7T and 21 min 36 s at 3T.

### Image analysis – qualitative assessment before and after rupture creation

All MRIs were reviewed for image quality and for type of finger flexor pulley injury by two independent musculoskeletal radiologists with 12 years, respectively 15 years of experience (T.B. and F.R.). Scoring of MRI images was blinded to the reports of the artificial injury induction and to the macroscopic analysis. Visualization of each finger flexor pulley (A2, A3 and A4) was evaluated according to a 4-point Likert scale from insufficient to excellent: 0 – insufficient: visibility of pulley heavily degraded due to insufficient spatial resolution, signal intensity or artifacts with no information about the pulley; 1 – poor: visibility of pulley degraded due to limitation of spatial resolution, signal intensity or disturbance by artifacts with poor information about the pulley visibility; 2 – good: sufficient spatial resolution and signal intensity with only slight artifacts resulting in a visualization of normal pulleys > 90% of their circumference; 3 – excellent: sufficient spatial resolution and signal intensity without disturbance by artefacts resulting in clear and sharp depiction of entire normal pulley. After artificial pulley rupture induction, the presence of an indeed created pulley rupture was also evaluated by both radiologists for each A2, A3 and A4 finger flexor pulley at 3T and 7T. Rupture patterns were determined as following: grade 0 – intact, grade 1 – rupture with adjacent stump on the flexor tendons, grade 2 – rupture with intercalation (dislocation of a ruptured pulley stump in between the flexor tendon and finger phalanx), grade 3 – rupture without visible stump and grade 4 – not assessable. A consensus reading for setting a matching evaluation of the presence or absence of a finger flexor pulley rupture was performed in cases of discrepancy between both radiologists.

### Macroscopic analysis

After MRI scans finger flexor pulleys were anatomically prepared by a specialized orthopedic surgeon with 5 years of experience (C.L.) in finger and wrist surgery and inspected for integrity or injury using magnifying glasses. Rupture patterns were determined analogous to radiological assessment by the orthopedic surgeon and recorded by photo documentation.

### Statistical analysis

Statistical analysis was performed using SPSS version 21.0 (SPSS Institute, Chicago IL, USA) for Windows® (Microsoft, Redmond, WA, USA). Kappa-statistics were used to calculate the interobserver agreement for the qualitative MR image analysis. According to Landis and Koch kappa-values of 0.41–0.60, 0.61–0.80, and 0.81–1.0 were considered to indicate moderate, substantial and almost perfect interobserver agreement, respectively [19]. Wilcoxon rank tests were used to evaluate for statistically significant differences between the two different field strengths with respect to the visibility of A2, A3 and A4 finger flexor pulleys (*P*-value < 0.05) [20]. Sensitivity, specificity, positive and negative predictive values of detected finger flexor pulley ruptures were calculated for 7T scans, respectively 3T scans.

## Results

Thirty cadaver fingers from ten paired hands were obtained from 3 female and 2 male donors for baseline MRI scan and for preparation. Three fingers of one left hand had to be excluded after the first MRI scan, due to complications with the freezing and un-freezing procedure. Consequently 90 finger flexor pulleys (A2, A3 and A4) prior to intervention and 81 pulleys after the mechanical loading process were assessed.

### Macroscopic analysis

In total 22 finger flexor pulley ruptures were induced in 10 fingers. The resulting rupture patterns were triple rupture (A2/A3/A4) in *n* = 5, combined (A2/A3) rupture in *n* = 1, single rupture (A2) in *n* = 3 and single rupture (A4) in *n* = 2 of the cases. Ruptures were localized at the left and right hands in equal parts. Ruptures occurred at the index finger in *n* = 13, at the middle finger in *n* = 5 and at the ring finger in *n* = 4 of the specimens. Finger flexor A2-, A3- and A4-pulley lesions were detected at the radial and ulnar, as well as in the central parts of the finger pulley in 33.3% each. 62.5% of all pulley lesions showed a dislocation and intercalation of the pulley stump in between the flexor tendon and finger phalanx.

### Image analysis

**Fig. 1:**
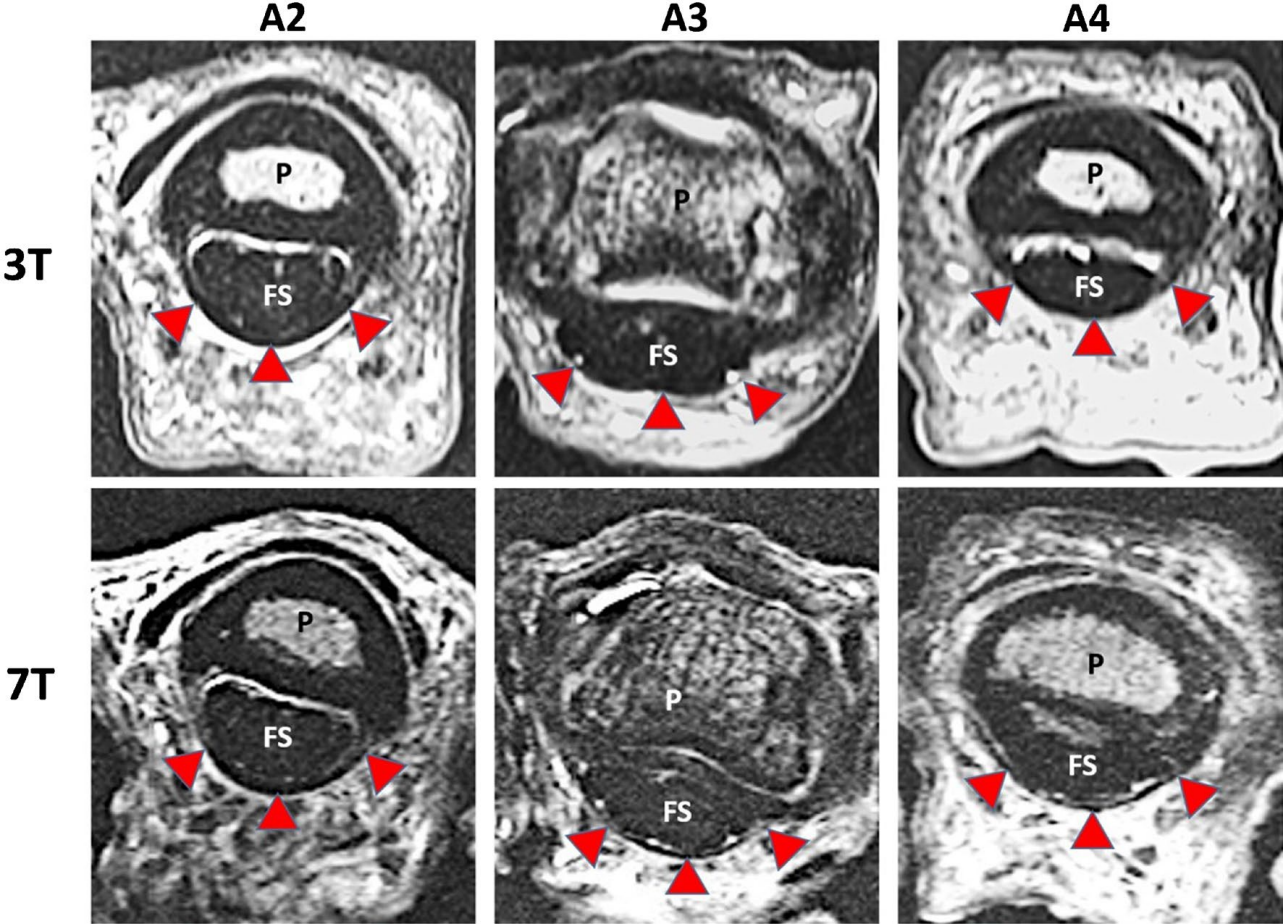
Axial PD sequence of the A2 (left), A3 (center) and A4 (right) pulley ligament before rupture at 3T (top) and 7T (bottom). P = phalanx, FS = flexor tendon, arrowheads = intact pulley ligament

**Fig. 2:**
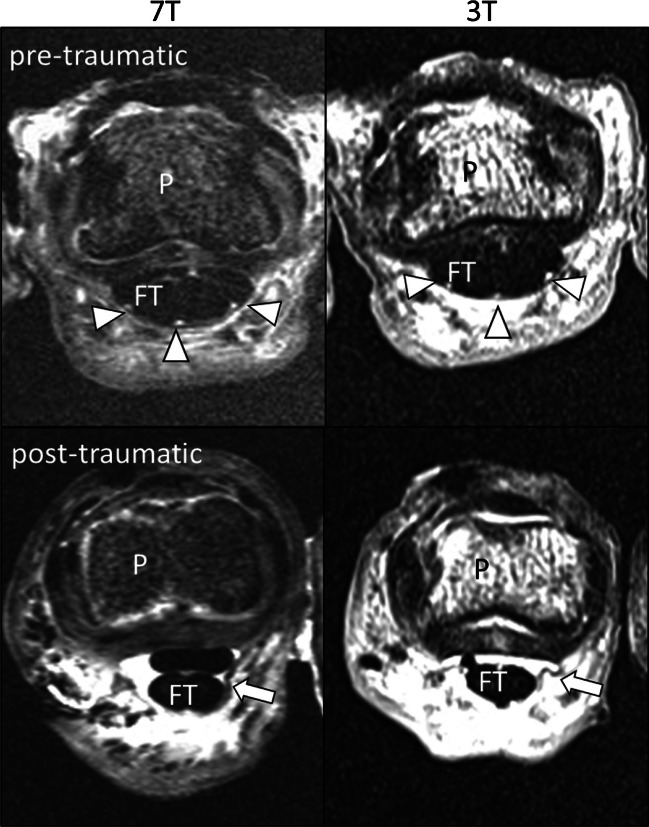
7T (left) and 3T (right) PD transverse scans before (above) and after (below) iatrogenic pulley rupture creation at A3 finger flexor pulleys. Intact and ruptured A3 finger flexor pulleys can be visualized at both field strengths. Arrowheads = intact A3 pulley. P = phalanx. FT = flexor tendon. Short arrow = disrupted pulley stump

**Fig. 3:**
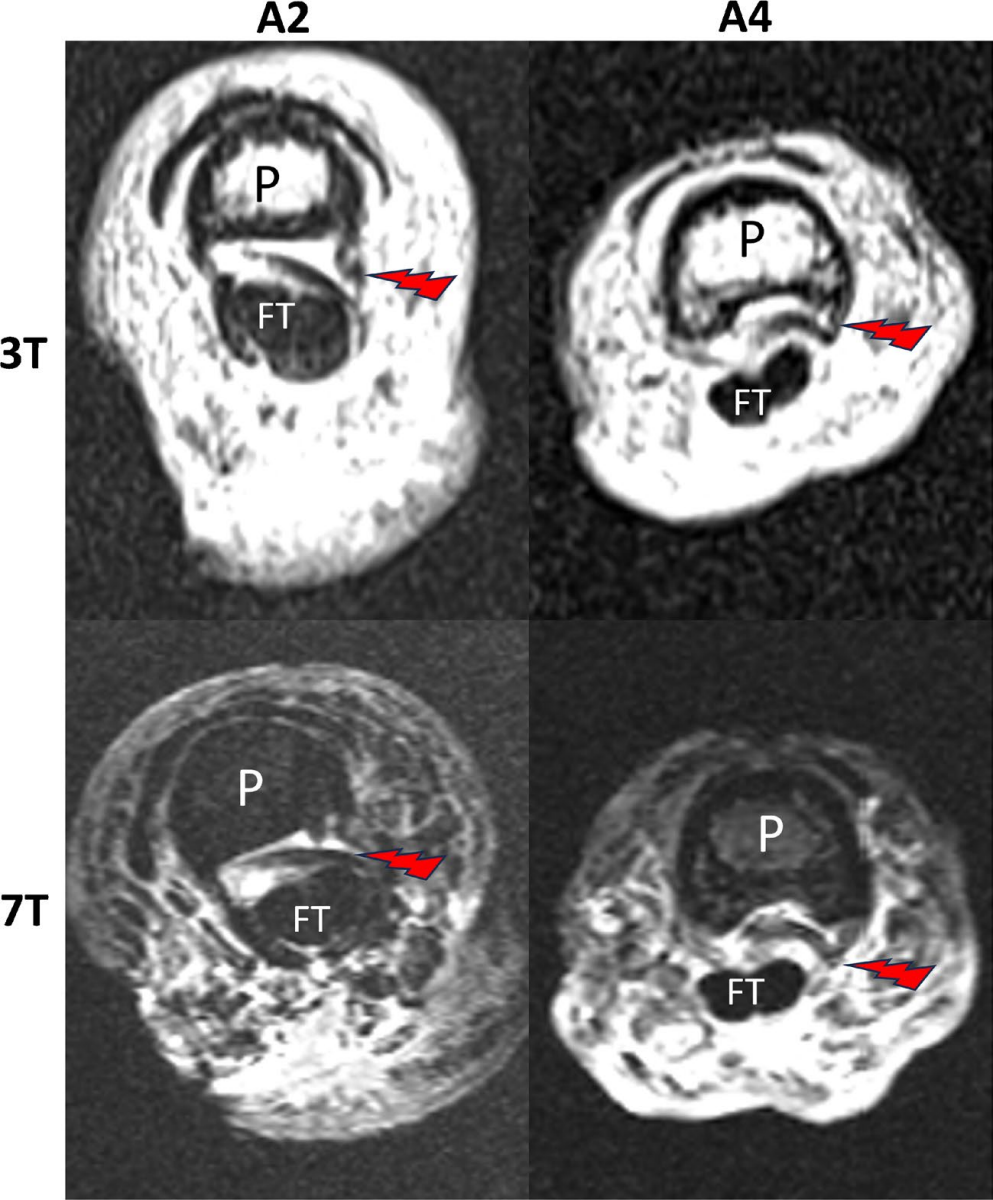
3T (above) and 7T PD transverse scans after iatrogenic pulley rupture creation showing pulley stump intercalations as rupture complication for the A2 pulley (left) and A4 pulley (right). Note the intercalation of the pulley stump in between flexor tendon and phalanx. Arrow = intercalated pulley stump. P = phalanx. FT = flexor tendon

In comparison of 7T and 3T sensitivity and specificity for the detection of A2, A3 and A4 pulley lesions were comparable with 100% vs. 95%, respectively 98% vs. 100%. In the assessment of A3 pulley lesions sensitivity of 7T was superior to 3T MRI (100% vs. 83%), whereas specificity was lower (95% vs. 100%). This resulted in a positive predictive value of 0.96 and a negative predictive value of 1.0 at 7T and a positive predictive value of 1.0 and a negative predictive value of 0.98 at 3T. Figures 1 and 2 show comparisons of the field strength 3 T and 7 T before and after rupture for the pulley ligaments. Corresponding comparisons for pulley stump intercalations as rupture complication are shown in Fig. 3.

The average Likert score for direct visualization of finger flexor pulleys before rupture was 2.7 and after rupture creation 2.8 at 7T, respectively 2.5 and 2.8 at 3T, reflecting adequate image quality in average at both field strengths.

The image quality due to the visibility of the A3 finger flexor pulley before rupture creation was significantly better for 7T compared to 3T scans (*p* < 0.001). The visualization of all other finger flexor pulleys before and after rupture creation was not enhanced at 7T when compared with 3T (Tables 1 and 2). Qualitative image analysis showed substantial interobserver agreement with overall kappa values of 0.9 and 0.8 for 7T and 3T, respectively.

**Table 1:** Visualization of each finger flexor pulley (A2, A3 and A4) according to a 4-point Likert scale (0–3) at 3T and 7T prior to artificial pulley injury induction

		A2	A3	A4
3 T	Reader 1	2.83	1.87	2.77
	Reader 2	2.50	1.87	2.77
7 T	Reader 1	2.77	2.70	2.67
	Reader 2	2.67	2.57	2.60
*p*-value	Reader 1	0.317	< 0.001	0.180
	Reader 2	0.059	< 0.001	0.070

**Table 2:** Visualization of each finger flexor pulley (A2, A3 and A4) according to a 4-point Likert scale (0–3) at 3T and 7T after artificial pulley injury induction

		A2	A3	A4
3 T	Reader 1	2.81	2.63	2.93
	Reader 2	2.70	2.63	2.93
7 T	Reader 1	2.93	2.90	2.97
	Reader 2	2.67	2.80	2.80
*p*-value	Reader 1	0.180	0.080	0.564
	Reader 2	0.405	0.157	0.102

## Discussion

MRI at 3T enabled direct visualization of A2, A3 and A4 finger flexor pulleys with a comparable diagnostic performance to 7T. Both field strengths had similar, almost perfect sensitivity and specificity for direct pulley rupture diagnosis. However, there was a superiority of subjective 7T image quality in pre-trauma direct characterization of A3 finger flexor pulleys. Imaging at both field strengths used a clinically applicable scan protocol of less than 22 min duration, which allowed for robust direct imaging. The subjective observer grading reflected comparable high image quality for both, 3T and 7T. Anatomical preparation served as reference standard in this ex vivo study.

Finger flexor pulleys are challenging to visualize due to the small anatomical size, especially the A3 pulley [8, 15]. In theory, ultrahigh-field MRI at 7T suggests superior visualization of small anatomical structures compared to lower field-strength MRI and may enable diagnosis of pathology previously not assessable [11, 15]. Hauger et al. and Bencardino described that for finger pulley imaging, the transverse plane proved to be more reliable than the sagittal plane and offered optimal visualization of the bony insertions of the pulleys [8, 21]. Therefore, only the transverse plane was included for direct imaging in our study protocol as described before [7, 15]. Previous studies emphasized that the theoretical SNR benefit at 7T does not necessarily translate into improved visualization of anatomical structures, which was also reported by Nordmeyer-Massner et al. or Heiss et al. comparing wrist MRI at 3T and 7T [20, 22]. This may partly be caused due to slightly stronger T2* blurring and increased fat-shift at 7T [20].

Our study was based on a clinical protocol, which was set up at 3T and adapted for 7T for direct comparison without considering special sequence adjustments based on the field strength. High-resolution imaging of the pulley ligaments requires thin slice thickness and a robust MR signal, which resulted in long acquisition times, especially for the turbo spin echo sequences at 3T. Compared to 3T, the stronger MR signal at 7T allowed for a 4-min reduction in the overall acquisition time. A further improvement in image quality based on SNR advantages through sequence optimization and specific coil adjustments is more likely in the future for 7T than with 3T, due to the lower degree of optimization achieved for 7T at current. For instance, dedicated, commercially available hand and finger coils are not yet available for 7T, unlike 3T. Nevertheless, further optimization potential can be assumed for both field strengths in the future, which should be examined in subsequent studies. For this perspective, the observed SNR gain will potentially be of key importance because it offers greatly enhanced latitude for protocol optimization [20]. In particular, it provides the possibility of reducing scan times, for example, by performing fewer averages or parallel imaging, and enhancing the spatial resolution [20].

In a clinical context, it is to be expected that direct pulley imaging approach as described in our study will become more and more relevant in the future. Due to the increasing popularity of the new Olympic climbing discipline pulley lesions will have a greater significance for healthcare systems [1]. Various diagnostic approaches have been developed in recent years [9, 23, 24]. Based on this, different therapeutic concept can now be offered, depending on the severity of the injury [1, 5, 25, 26]. Partial or complete rupture of a single pulley is commonly treated conservatively, and complete ruptures of multiple pulleys are preferably treated surgically 1,5,23,24]. Therefore, precise imaging definition of the injury pattern is requested by surgeons, for optimized therapy planning. Also, complications of pulley ruptures, such as residual pulley stump dislocation, can now be detected with direct imaging presurgically, and then treated in a targeted manner [15]. Clinical examination, radiographs and computed tomography cannot point out these specific pulley injury patterns and complications, but will rather demonstrate a non-specific soft tissue swelling beside the “bowstring sign” [27]. The same applies for bowstring assessment with US and/or 1.5T MRI, which may also not show the full spectrum of injuries compared to the direct high-field and ultra-high-field imaging techniques mentioned here. A study by Schöffl and al. with high-resolution US was able to directly visualize the A3 pulley in 61% of cases [9]. Further studies on direct US evaluation of the pulleys, including detection of complications such as stump dislocation or graduation of partial ruptures, are pending. Therefore, our results suggest, that in case of suspected pulley rupture, further MRI diagnostics with a direct visualization approach including the functional important A3 and A4 pulley [28] may be beneficial, which can be performed with a field strength of 3T or 7T without a functional examination or stress during finger positioning.

Our study has several limitations. Findings of a cadaver study cannot be directly translated to an in-vivo setting, because of potentially changed biomechanical characteristics of post-mortal tissue and the artificial lesion preparation. Secondary lesion signs, such as edema or local hemorrhage, which may facilitate the detection in clinical practice, were absent. Because of the artificial induction of pulley ruptures, air entrapment between tendon and bone occurred in some specimen, obscuring the image quality. Due to the experimental study setup, the incidence of triple pulley rupture and of complications such as stump intercalation, were higher than expected clinically. It needs to be stressed that the fingers included were from an elderly population, which is in contrast with the general mean age of active climbers. An important further limitation is the use of different MRI coils at 3T and 7T, which means an additional variable besides the two field strengths.

## Conclusion

MRI at 3T allows direct visualization of A2, A3 and A4 finger flexor pulleys with a comparable diagnostic performance to MRI at 7T. There was a superiority of subjective 7T image quality in pre-trauma direct characterization of A3 finger flexor pulleys. In a clinical context, direct imaging using 3T and 7T installations may have the potential to improve diagnostic confidence in patients with suspected finger flexor pulley injuries, without the need for stress or functional imaging. This may be beneficial for guiding appropriate treatment strategies with improved clinical outcomes.
